# Impact of Trade Openness on Food Security: Evidence from Panel Data for Central Asian Countries

**DOI:** 10.3390/foods10123012

**Published:** 2021-12-05

**Authors:** Zhilu Sun, Defeng Zhang

**Affiliations:** Institute of Agricultural Economics and Development, Chinese Academy of Agricultural Sciences, Beijing 100081, China; 17863905481@163.com

**Keywords:** food security, trade openness, countries of Central Asia, sustainable food system

## Abstract

The problem of food insecurity has become increasingly critical across the world since 2015, which threatens the lives and livelihoods of people around the world and has historically been a challenge confined primarily to developing countries, to which the countries of Central Asia, as typical transition countries, cannot be immune either. Under this context, many countries including Central Asian countries have recognized the importance of trade openness to ensure adequate levels of food security and are increasingly reliant on international trade for food security. Using the 2001–2018 panel data of Central Asian countries, based on food security’s four pillars (including availability, access, stability, and utilization), this study empirically estimates the impact of trade openness and other factors on food security and traces a U-shaped (or inverted U-shaped) relationship between trade openness and food security by adopting a panel data fixed effect model as the baseline model, and then conducts the robustness test by using the least-squares (LS) procedure for the pooled data and a dynamic panel data (DPD) analysis with the generalized method of moments (GMM) approach, simultaneously. The results show that: (1) a U-shaped relationship between trade openness and the four pillars of food security was found, which means that beyond a certain threshold of trade openness, food security status tends to improve in Central Asian countries; (2) gross domestic product (GDP) per capita, GDP growth, and agricultural productivity have contributed to the improvement of food security. Employment in agriculture, arable land, freshwater withdrawals in agriculture, population growth, natural disasters, and inflation rate have negative impacts on food security; and (3) this study confirms that trade policy reforms can finally be conducive to improving food security in Central Asian countries. However, considering the effects of other factors, potential negative effects of trade openness, and vulnerability of global food trade network, ensuring reasonable levels of food self-sufficiency is still very important for Central Asian countries to achieve food security. Our research findings can provide scientific support for sustainable food system strategies in Central Asian countries.

## 1. Introduction

The problem of food insecurity has become increasingly critical across the world since 2015, which threatens the lives and livelihoods of people around the world, particularly the most vulnerable classes [1,2]. According to the latest estimates, at the global level, it is projected that in 2020, between 720 million and 811 million people suffered from hunger, and nearly 2.37 billion people did not obtain enough food, considering the median value, as many as 161 million and 320 million people more than in 2019, respectively [2]. At the same time, all regions of the world will be affected, taking Central Asia for example, in 2020, 2.6 million people were suffering from hunger, being 18.18% higher than in 2019 and one of the regions with a significant growth rate [2]. The COVID-19 pandemic—that broke out at the end of 2019—has further aggravated current trends and policy reactions have triggered a massive economic recession and major disruptions in national and global food supply chains [3,4,5,6], and the World Food Programme (WFP) estimated that 272 million people have already been or will soon become acutely food insecure in countries where the COVID-19 pandemic is present [7]. The World Food Summit in 1996 set a goal for halving undernourished populations all around the world by 2015, being one of the eight Millennium Development Goals (MDGs) [8]. In order to offer a new global approach for building sustainable food and nutrition system from 2015 to 2030, the 17 Sustainable Development Goals (SDGs) built on the MDGs, were adopted at the Sustainable Development Summit in 2015, among which the SDG2 is aimed at ending hunger, improving food security status, and promoting sustainable agriculture and food [9]. Therefore, in recent years, the world has not been generally progressing towards the SDG2, making it more difficult to realize the Zero Hunger goal by 2030, so does the Central Asia region.

According to the Food and Agriculture Organization (FAO) of the United Nations (UN), the most widely used and authoritative definition of food security today is described as a condition in which all people, at all times, have physical, social, and economic access to sufficient, safe, and nutritious food that meets their dietary needs and food preferences for an active and healthy life [10,11,12]. Therefore, based on this perspective, food security means regularly having enough food to eat, not just for today or tomorrow, but also for the next month and next year, and is a global issue affecting every human being’s daily life, both in developing and developed countries [13,14]. The FAO suggests food security is generally the composite of four pillars, including availability, access, stability, and utilization [15], based on which food security could be better understood when presented through a series of indicators corresponding to each pillar; furthermore, it is possible to conduct a scientific assessment of the food security status from the local level to the global level.

The phenomenon of food insecurity has historically been an issue confronted primarily by developing countries [16], where millions of people are suffering from serious undernourishment due to food insecurity [14], and the transition countries cannot be spared either. After independence from the Soviet Union, five Central Asian countries—Kazakhstan, Kyrgyzstan, Tajikistan, Turkmenistan, and Uzbekistan, underwent a series of transitions from centrally planned systems to market-orientated economies [17]. However, after independence, due to a number of challenges, such as continuing economic instability and household incomes decline, poor design and slow implementation of economic and political reforms for reorienting of agricultural production from collective farms to private farms, inefficient agricultural infrastructure, and low levels of commercial agricultural inputs [17,18,19], food insecurity remained at a high level in Central Asian countries, then great concerns over food security emerged, therefore, achieving food security is at the forefront of these countries’ economic strategies and policies [20,21]. Despite minor positive adjustments at the break of this century, the agricultural sector in most Central Asian countries was hit hard yet again by the 2007–2008 food price crisis, which further aggravated the food insecurity problem in the region [22]. In recent years, Central Asian countries have experienced several transitions in institutions and governance, each with consequences for agriculture in the region, and have made considerable progress towards increasing domestic food production [23,24], but food security in the region is still threatened by a series of geopolitical, socio-economic and environmental challenges, such as lack of support from institutions to advance farming, lack of efficient and reliable transport systems and adequate infrastructure, lack of research and extension of support to farmers, low capacity for designing evidence-based policies, small-scale farming of land use, and land degradation aggravated by climate change [25,26,27], contributing to limited productivity in agriculture which is unable to fully satisfy domestic food demand, especially in Tajikistan and Kyrgyzstan both of which are highly dependent on the import of basic food commodities [28,29].

Using 2001–2018 panel data for Central Asian countries, from the four pillars of food security, including availability, access, stability, and utilization, this research empirically estimates the effect of trade openness and other influences on food security by performing the following work. First, we empirically estimate the effect of trade openness and other influences on food security and trace a U-shaped (or inverted U-shaped) relationship between trade openness and food security by adopting a panel data fixed effect model as the baseline model. Second, we conduct a robustness test by using the least-squares (LS) procedure for pooled data and a dynamic panel data (DPD) analysis with a generalized method of moments (GMM) approach, simultaneously. Then, this study makes policy implications for improving the food security status in Central Asian countries based on research findings.

## 2. Literature Review

Food security is traditionally analyzed in terms of food self-sufficiency or food self-reliance [30]. Self-sufficiency requires domestic food consumption mainly depends on domestic production rather than imports as the main supply source, while self-reliance requires more diverse supply sources, including imports [30,31]. In recent years, international food trade has become an increasingly integral part of the global food supply system [32], and more countries have recognized the significance of trade openness to maintain adequate food security status [14] and are increasingly dependent on trade to maintain food security due to climate change, population growth and many other factors. It is estimated that one in every six people around the world presently depends almost entirely on international trade for the food they eat, a proportion that could rise to 50% by 2050 [33].

In light of the fact that trade openness plays an important role in each country’s food security, the effect of trade openness on food security deserves deep consideration. However, long-standing debates over this issue remain unresolved [12], and it is found in the relevant literature that the impact is mainly twofold. On one hand, based on the trade policy reform from the World Trade Organization (WTO) multilateral negotiations, and regional and bilateral trade negotiations, deterministic interests from trade attained through the removal or reduction in tariff and nontariff barriers to trade and relaxation of market access is conducive to making up for the shortages of the domestic food supply by increasing the accessibility to the international market and reducing the price of imported goods, and improving the availability and diversity of foods [2,30,34,35,36]. The WTO and World Bank are continuously promoting the concept that international market access plays an important role in its clients’ development, especially developing countries, and is increasingly attracting attention for the removal of trade restrictions mainly imposed by developed countries to developing countries [37]. With adequate domestic social safety-net policies for vulnerable classes, trade openness has been consistent with food security by lowering food price which benefits everyone including those suffering from most serious food insecurity, and a more diversified diet has made poor classes less vulnerable to food unavailability or price fluctuation in specific foods [38]. Although tariffs have increased the spread of global price fluctuation and exposed consumers to momentary price spikes, the duration of which is likely to be short, unlike the time with low prices may be endured, but also allowed them to benefit from extended periods of low prices [2]. Therefore, trade openness would increase host countries’ economic welfare and decrease its economic volatility [39], especially the protection of domestic agricultural market would harm, rather than ensure their food security, which has been observed in many countries [34,38].

On the other hand, stochastic gains and losses caused by supply-side impacts such as fluctuating agricultural productivity, violent conflict, and war, natural disasters including drought and embargoes can be magnified or reduced depending on trade openness level, and can endanger food security [40]. Trade openness has lowered the level of food self-sufficiency, and has made the food supply more dependent on imports [34], which is supposed to make the food supply less secure, especially considering that agriculture is incompatible with free trade because of its innate role in managing ecological/natural resources at both national and global levels and the uneven playing field that was created from how agriculture has been treated (protected/taxed) differently across countries [41,42]. Liberalizing trade, especially in agriculture, is not conducive to unleashing agricultural production potential, considering the market competitiveness of smallholder farmers in developing economies is significantly weaker than large farmers in middle-income economies or subsidized farmers in developed economies [41]. Additionally, reducing foreign dependency on food could significantly abate the conveyance of international market shocks to indigenous markets [31]. It has also been found in some relevant literature that there was a U-shaped relationship between trade openness and food security in less developed countries (LDCs), and food security has decreased in the initial stages of trade openness expansion but increased beyond a given threshold [43].

The relationship between trade openness and food security is likely to provide the empirical evidence necessary to address and respond to one of the key issues during the WTO’s international negotiations, and regional or bilateral trade negotiations [12,38], although the prospects for positive and beneficial solutions are still uncertain [44]. According to the relevant literature, various factors can affect trade openness’s impact on food security, especially considering that food security is a multipillarsal issue, and make it difficult to determine whether trade openness’s impact on food security is positive or negative for specific countries only by qualitative and descriptive analysis performed by the majority of previous studies [12,36,45]. As typical transition economies, Central Asia represents an important strategic junction and its economic development has important connotations for Central and Eastern Europe, China, and the Middle East and West Asia, and improving food security is vital for sustainable economic development in Central Asia [17]. However, quantitatively empirical studies that assess trade openness’s potential impact on food security in Central Asian countries are still rare. An additional problem is that most previous studies used poverty indicators rather than direct food security indicators for representing food security [14,36].

Therefore, this study aims to quantitatively explore trade openness’s impact on food security in Central Asian countries. Using four different direct food security indicators that correspond to the four pillars of food security, including availability, access, stability, and utilization, respectively, a panel data fixed effect model has been established to empirically analyze trade openness’s impact on food security in Central Asian countries. We explore the following questions: (1) Is trade openness a threat or an opportunity for food security in Central Asian countries? Is there a U-shaped (or inverted U-shaped) relationship between trade openness and the four pillars of food security? (2) Do other economic and non-economic factors chosen as control variables have positive or negative effects on food security in Central Asian countries?

## 3. Materials and Methods

### 3.1. Methods

#### 3.1.1. Baseline Model

As indicated previously, this study aims to empirically analyze trade openness’s impact on food security in Central Asian countries by adopting a panel data fixed effect model as the baseline model. Considering the trade openness–food security relationship and the effect of other important potential determinants, and based on the model developed by Kang [43], Dithmer and Abdulai [36] and Fusco et al. [14], we built the panel data fixed effect model as:

(1) $$FS_{i,t}=\alpha_{0}+\alpha_{1}TO_{i,t}+\alpha_{2}TO_{i,t}^{2}+\alpha_{3}CV_{i,t}+T_{i,t}+C_{i,t}+\varepsilon_{i,t}$$

where *i* and *t* denote Central Asian countries and time periods, respectively.

As the dependent variable, *FS* represents food security and is denoted by following four indicators that respectively correspond to the four pillars of food security, including availability, access, stability, and utilization, considering the data availability across Central Asian years and countries of all food security indicators in the Food and Agriculture Organization Corporate Statistical Database (FAOSTAT; http://www.fao.org/faostat/en/#data/FS (accessed on 16 March 2021)): (1) dietary energy supply, used as a proxy for availability, which is computed by comparing a probability distribution of habitual daily dietary energy consumption with the minimum dietary energy; (2) rail lines density, used as a proxy for access, corresponds to the ratio between the length of railway route available for train service, irrespective of the number of parallel tracks within the country; (3) food supply variability, used as a proxy for stability, represents the per capita variability of the “food supply in kcal/caput/ day” as disseminated in the FAOSTAT; (4) population using safely managed drinking water services, used as a proxy for utilization, corresponds to the ratio between population using safely managed drinking water services and total population. These four pillars are necessary for food security to be understood as a continuum that progresses from uncertainty and anxiety regarding access to sufficient and appropriate food at the household level to the extreme condition of hunger among children because they do not have enough to eat [46].

$TO_{it}\;$is the trade openness measure, which is computed as the share of trade value (the sum of exports and imports of goods and services) over the gross domestic product (GDP) from the World Bank’s World Development Indicators (WDI; https://data.worldbank.org/indicator?tab=all (accessed on 16 May 2021)). This trade openness measure is the measure usually employed in impact studies of trade openness and is arguably better than de jure measures (e.g., tariffs) to the extent that the latter are difficult to summarize in a single indicator [36] and has great advantages in that it can reflect the entirety of a country’s commercial transactions, including exports and imports [47]. $TO_{i,t}^{2}\;$is the quadratic form of$\;TO_{it}$, which is also treated as an independent variable for testing whether there is a U-shaped (or inverted U-shaped) relationship. If $\alpha_{2}$ is statistically significant, then it is demonstrated as a U-shaped (or inverted U-shaped) relationship. *CV* is a set of control variables that might be major determinants in Central Asian countries’ food security and are explained in more detail below. $T_{i,t}$ and $C_{i,t}$ are the period fixed effects (such as geographic characteristics or unobserved cultural and institutional factors) and cross-section fixed effects (such as changes in world prices and shocks that are identical to all countries), respectively; $\alpha_{0}$ is the constant term; $\alpha_{1}$, $\alpha_{2}\;$and $\alpha_{3}$ are the estimated values of independent variables’ coefficients; and $\varepsilon_{i,t}\;$is the random error term.

#### 3.1.2. Robustness Analysis Models

For verifying the validity of panel data fixed effect model’s estimation results, we used an LS procedure for pooled data and a DPD analysis with the GMM approach, simultaneously.

(1) LS procedure for pooled data

The estimation equation form of the LS procedure for pooled data is:

(2) $$FS_{i,t}=\beta_{0}+\beta_{1}TO_{i,t}+\beta_{2}TO_{i,t}^{2}+\beta_{3}CV_{i,t}+\varepsilon_{i,t}$$

where *β*_0_ is the constant term; and *β*_1_, *β*_2_ and *β*_3_ are the estimated values of independent variables’ coefficients.

(2) DPD analysis with GMM approach

Considering the potential effect of past food security levels and testing for the dynamic effects and the endogeneity problem, based on Nickell [48], Holtz-Eakin et al. [49], and Arellano and Bond [50], the estimation equation form of DPD analysis with the GMM approach is:

(3) $$FS_{i,t}=\phi_{1}FS_{i,t-1}+\phi_{2}TO_{i,t}+\phi_{3}TO_{i,t}^{2}+\phi_{4}CV_{i,t}+T_{i,t}+C_{i,t}+\varepsilon_{i,t}$$

where $FS_{i,t-1}\;$ is the lagged food security and treated as predetermined to the current period for modeling current food security levels as a function of past food security levels (also capturing the effects of past status), and current determinants [36]. The lagged dependent variable would control for longer-term effects of all our included control variables and would also pick up effects of other potentially omitted variables as it will be highly correlated with the current values of the food security variables [14,36]. *ϕ*_1_, *ϕ*_2_, *ϕ*_3_ and *ϕ*_4_ are the estimated values of independent variables’ coefficients.

### 3.2. Data

Based on the FAO’s conceptual analysis framework for trade policy reforms and food security [11,38], experiences of previous literature, and data availability, we used panel data composed of five Central Asian countries over the period 2001–2018. Most of the data used in this study come from the FAO’s FAOSTAT and World Bank’s WDI. According to FAO [11,38], Dithmer and Abdulai [36], and Fusco et al. [14], we considered the following five categories of food security determinants: the first category is trade openness measure; the second category accounts for country context and characteristics; the third category describes the development of economy and population; the fourth category captures non-economic events; the fifth category denotes domestic macroeconomic policies except trade policy. The trade openness measure, as the first category, is the core independent variable in this study and has already been explained in detail earlier. Subsequently, we focus on the last four groups of food security determinants, which represent the control variables included in Equations (1)–(3).

Referring to the second category, we take into account the following three aspects to represent the country’s context and characteristics: macroeconomic scale, importance of agriculture, and availability of resource endowment for agricultural production in the country. Gross domestic product (GDP) per capita in constant 2010 USD is used as the variable to measure the macroeconomic scale and data on which comes from the WDI. A positive impact of GDP per capita on food security is projected, as strong income growth is crucial to enhance the acquisition for buying food [36]. The importance of agriculture is measured by the employment in agriculture, which refers to the share of persons of working age who were engaged in the agricultural industry of total employment, and data on which comes from the WDI. The first important element of national resource endowment for agricultural production is arable land per person and data on which comes from the WDI. In the context of agricultural land use, the function to produce biomass is the most relevant to sustain food production and respond to cultural practices conducive to sustainable agricultural land management [51]. The second important element of national resource endowment for agricultural production is freshwater withdrawals in agriculture, which refers to annual freshwater withdrawals in agriculture divided by total freshwater withdrawal, and data on which comes from the WDI. The amount and timing of water available for irrigation have a direct effect on crop yields, which is vital for ensuring food production, and water security is the basis for food security [52,53].

Referring to the third category, three variables are used to reflect the agricultural and overall development of the economy and population: agricultural productivity, GDP growth, and population growth. Cereal yield is used as a proxy for agricultural productivity, and data on which comes from the WDI. Despite increasing internationalization, many developing countries’ food security still mainly depends on domestic agricultural production [54], then agricultural productivity growth is one of the important approaches to enhance domestic food availability and decrease their prices [55,56]. GDP growth is used to capture cyclical fluctuations in total output, and data on which comes from the WDI. Increases in GDP are associated with increasing income per capita and changes in demands, often reflected in stronger demand for food and resulting in increased consumption [57]. Population growth is used to describe population pressures on food security, and data on which comes from the WDI. Population pressures as denoted by the rapid population growth rate always result in increasing food demands for the total population and reduce food availability per capita [36].

Referring to the fourth category, one variable is chosen to represent non-economic events: natural disasters. The natural disasters data are from the Emergency Events Database (EM-DAT; https://public.emdat.be/ (accessed on 28 May 2021)) of Belgium launched by the Centre for Research on the Epidemiology of Disasters (CRED), which is calculated by the ratio between the total number of injured, affected, and homeless populations as a direct result of natural disasters (including geophysical disasters, meteorological disasters, hydrological disasters, climatological disasters, biological disasters, and extra-terrestrial disasters) and total population. When natural disasters affect the agriculture sector, they can have far-reaching negative consequences beyond physical damage, they: lower production and productivity; decrease exports of agricultural commodities, and increase food imports, causing disequilibrium in the balance of trade and in the balance of payments in affected countries; and arrest agriculture sector growth and the sustainable development of the sector [58].

Referring to the fifth category, one variable is chosen to capture domestic macroeconomic policies except for trade policy: inflation rate (GDP deflator). The inflation rate (GDP deflator) calculated by the annual growth rate of GDP implicit deflator, denotes the price’s changing rate at a national level, and data on which comes from the WDI. The inflation rate is an important indicator of macroeconomic stability [59,60]. Considering national food security, macroeconomic policies that affect certain vital macroeconomic factors can have both a direct and indirect impact on a country’s rate of food production and food inflation [61], and high efficiency in development and economic growth will be conducive to reducing poverty and hunger and finally eliminating food insecurity in every country [62].

The variables and their data sources, and their descriptive statistics used in this study are presented in Table 1 and Table 2, respectively.

### 3.3. Descriptive Analysis on the Food Security Status in Central Asian Countries

**Figure 1:**
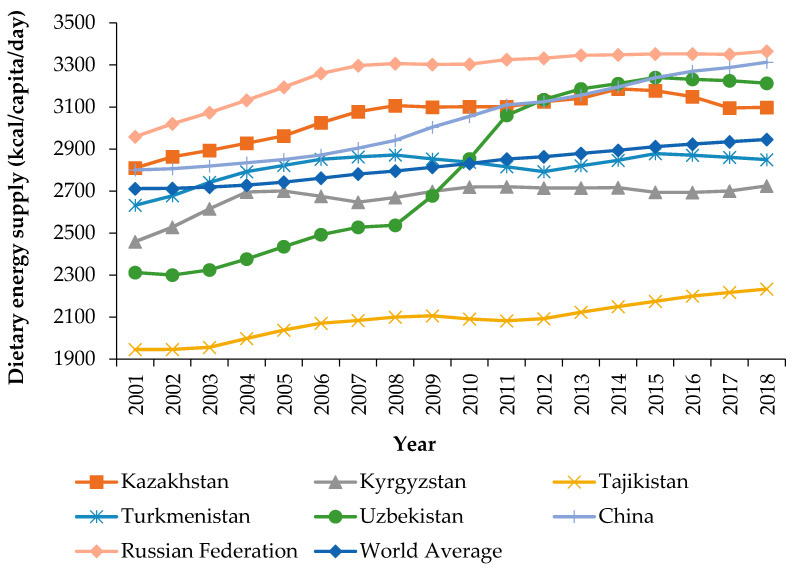
The “availability” pillar of food security in Central Asian countries, China, the Russian Federation, and the world average from 2001–2018. The x-axis represents the year. The y-axis refers to food security’s “availability” pillar, which is denoted by the dietary energy supply.

Taking the “availability” pillar of food security as an example, which is denoted by the dietary energy supply in this study, we first conducted a descriptive analysis of the evolutionary characteristics of food security status in Central Asian countries and also conducted a comparison with two major transition countries (China and the Russian Federation) and the world average. Then, we further briefly discussed the ongoing COVID-19 pandemic’s impact on food security status in Central Asian countries. Figure 1 shows the evolutionary trends of the dietary energy supply in five Central Asian countries, China, the Russian Federation, and the world average from 2001–2018.

After independence, Central Asian countries underwent a series of transitions from centrally planned economies to a market-orientated system [18]. During the initial years of transition, all the Central Asian countries experienced rising poverty, food insecurity, and malnutrition [18,63]. Therefore, Central Asian countries have attached great importance to improving their food security status by adopting food self-sufficiency policies, and for implementing such an approach; legislation including laws and strategies to support agricultural producers by various subsidies for input factors in production, such as fertilizers, seeds, plant protection pesticides, fuel and electricity, extension technicians, and agricultural machinery services have been developed [64]. Then, the dietary energy supply in most Central Asian countries totally showed positive trends in the 21st century. Land reform and farm restructuring also contributed to the improvement of food security in Central Asian countries. Kazakhstan and Kyrgyzstan were relatively faster in privatization reform of land. Taking Kazakhstan as an example, the share of non-state enterprises reached 95% of arable land and 91% of animal husbandry by 1999, and the private business had dominated the agro-industry [23,65]. In Uzbekistan and Turkmenistan, governments adopted slower paths for the transition to a market-orientated economy, and preserved state ownership and controlled most agricultural land, and land reform mainly focused on fragmentation of large collective and state farms but without transferring land ownership to farmers [23,66]. In Tajikistan, the civil war immediately after independence in 1992–1997 had strong damaging effects on its agricultural production, and privatization reform of land was initiated later than in other Central Asian countries [67].

Since 2007, the dietary energy supply in Kazakhstan has been the highest among Central Asian countries from 2001–2011, and Uzbekistan overtook Kazakhstan to become the highest from 2012–2018, while Tajikistan has always been the lowest. By 2015, as a region, Central Asia achieved MDGs 1C goal (halving the proportion of people suffering hunger from 1990–2015), but the progress on reducing hunger incidence differed from country to country. Kazakhstan achieved the MDG 1C goal as early as 2006; Kyrgyzstan, Turkmenistan, and Uzbekistan achieved the goal in 2014–2016; Tajikistan has consistently had the highest rate of undernourishment in the region (the percentage of the population with a caloric intake below the minimum dietary energy requirement). The prevalence of undernourishment in Tajikistan was estimated at 28.1 percent in 1990–1992, climbing to 33.2 percent in 2014–2016 [64], showing serious food insecurity issues. The year 2016 and beyond have marked the transition from the MDGs to the SDGs, which recognized the unfinished nature of efforts to eradicate poverty and hunger, but also expanded the coverage of programs to include both developing and developed countries and widen the range of the goals to include sustainability as a fundamental leitmotif. In line with Goal 2 of the SDGs, calling for the eradication of hunger, achieving food security, and the elimination of all forms of malnutrition, national authorities in Central Asia will build on progress made in the region in sharply improving food and nutrition security since 1990 by identifying their national priorities to achieve this goal [68]. However, since 2016, the dietary energy supply in Kazakhstan, Uzbekistan, and Turkmenistan has tended to decline—mainly due to insufficient quantities of vegetables, fruits, and fish. This trend has pointed to the urgent demand for more efforts towards raising the availability of several food items through nutrition-sensitive policies in both production and trade areas [69]. In 2018, the dietary energy supply in all Central Asian countries was still significantly lower than that in China and the Russian Federation, especially Tajikistan is only 67.5% and 66.4% of China and Russia Federation, respectively; when compared to the world average, only the dietary energy supply in Kazakhstan and Uzbekistan was higher than the former. Therefore, the food security status in Central Asian countries needs to be further improved, not to mention the nutrition issues in these countries, and larger efforts from within and outside the region need to be carried out comprehensively to eliminate food insecurity as soon as possible.

The COVID-19 pandemic—still ongoing all around the world as of 2021—is also present in Central Asia, which is further worsening the food insecurity status in Central Asian countries, in particular, it is estimated that the number of food-insecure people in Central Asia is expected to grow significantly, which will become worse as the COVID-19 continues to spread. A survey conducted by the FAO Regional Office for Europe and Central Asia reveals that COVID-19 has led to disruptions in transportation, storage, output deliveries, input supplies, and operational financing throughout the food supply chains and has continued to pose operational challenges that affect crop farmers, livestock farmers, and traders/processors in most countries in the region [70]. As COVID-19 has disrupted markets, food prices have risen, the incomes of many rural and urban families have fallen, especially low-income classes, and the diversity of food has decreased [4]. Moreover, COVID-19 has negatively impacted Central Asian countries’ food security, and the innutrition status is also likely to deteriorate, which will significantly heighten the difficulty of realizing the Zero Hunger goal on schedule by 2030 in Central Asia.

## 4. Results and Discussion

### 4.1. Estimation Results of Panel Data Fixed Effect Model

#### 4.1.1. Data Test

##### Correlation Analysis

Considering the possible multicollinearity issues in the models we have built, which could negatively affect the reliability of estimation results, a correlation analysis was computed between independent variables. According to the correlation analysis results presented in Table 3, we found that the vast majority of absolute values of the Pearson correlation coefficient between the independent variables were less than 0.5, then based on the determination criteria for the degrees of correlation and the values of Pearson correlation coefficient [71], we can confirm that there was no strong multicollinearity in the equations, therefore, we can continue to conduct the empirical analysis based on these equations.

##### Panel Cointegration Test

In the macroeconomic panel data, due to identical impacts or spillover effects, cross-sectional dependence would arise, and for which if not accounted for, biased estimated results may be obtained [72]. Therefore, since the early 2000s, aiming to analyze the existence or otherwise of long-run stationary relationships among integrated economic variables, several kinds of panel cointegration test methods have been developed [73], such as the Pedroni (Engle-Granger Based) cointegration test [74,75], the Kao (Engle–Granger Based) cointegration test [76], and combined individual tests [77]. In order to examine the relationship among the dependent variable (the selected four proxy variables corresponding to food security’s four pillars), core independent variable, and control variables in this study, the Kao cointegration test was employed here. Under the null hypothesis of no cointegration, the Kao cointegration test specifies cross-section-specific intercepts and homogeneous coefficients on the first-stage regressors [76]. When we conducted the Kao cointegration test, the individual intercept was selected for deterministic trend specification, lag length was automatically selected by using Schwarz information criterion (SIC), Bartlett kernel was selected for spectral estimation, and bandwidth was automatically selected by using Newey–West. According to the Kao cointegration test results presented in Table 4, we found all the values of the augmented Dickey–Fuller (ADF) t-Statistic were statistically significant at a 1% significance level, and all the values of probability corresponding to ADF t-Statistic were less than 1%. Therefore, the null hypothesis of no cointegration was strongly rejected, and long-run stationary relationships existed among the dependent variable, core independent variable, and control variables in this study.

#### 4.1.2. Estimation Results

The specification for the panel data fixed effect model has period and cross-section fixed effects, based on which three specifications could be estimated: one only with period fixed effects, one only with cross-section fixed effects, and one only with a common intercept. The redundant fixed effects test should be used to determine the optimal specification for the panel data fixed effect model, and specifically using the likelihood function (chi-square test). Cross-section chi-square is used to evaluate cross-section fixed effects’ significance, period chi-square is used to evaluate the period fixed effects’ significance, and cross-section/period chi-square is used to evaluate both of the effects’ joint significance, respectively. According to the redundant fixed effects test results presented in Table 5, all the three statistic values corresponding to the above three tests in columns (1)–(4) were statistically significant. Therefore, both null hypotheses suggest that cross-section fixed effects are redundant and no period fixed effects were strongly rejected, and the optimal specification of panel data fixed effect model in this study should include period and cross-section fixed effects, simultaneously. Additionally, considering that period and cross-section specific effects’ presence may also be handled by using random effect methods, this study further used the Hausman test to compare the results from the estimated random effect specification and a corresponding fixed effect specification, with the null hypothesis that the random effect is correlated with the right-hand side variables in the panel equation setting. Random effect estimation requires the number of coefficients for between estimator for an estimate of random effect innovation variance is less than the number of cross-sections [78], which is not applicable in this study, therefore, when we used random effect methods, cross-section random effects are excluded, and only period random effects are included. According to the Hausman test results presented in Table 5, all the statistic values were statistically significant, which means that the null hypothesis was strongly rejected, and fixed effect specification is more appropriate than random effect specification in this study.

Table 5 presents estimation results of the panel data fixed effect model which takes the selected four proxy variables corresponding to food security’s four pillars as dependent variables. The results in columns (1)–(4) show that *TO* has negative coefficients and *TO*^2^ has positive coefficients, and both are statistically significant. Therefore, the estimation results of the panel data fixed effect model show a U-shaped relationship between trade openness and food security’s four pillars in Central Asian countries, which is similar to the results of Kang [43], but different from the findings of Fusco et al. [14] and Dithmer and Abdulai [36]. The early stages of trade openness negatively impact food security, which implies that increased trade openness contributes to the redistribution of world production based on a comparative advantage due to trade and globalization [43,79]. Considering that trade openness means a change in relative prices of traded and non-traded commodities [38], the more a country becomes reliant on traded food, the more it will be inducting the “global food price” for related goods, and global inflation will more negatively influence its low-income groups, which spend most of their family income on food, resulting in increased food security risk [80], especially in transition economies such as Central Asian countries, where a perfect market-oriented economic system has not yet been established since achieving independence from the Soviet Union [24,81]. Additionally, benefits from a nation’s larger openness to food security might be neutralized by greater unsteadiness or ecological costs [82,83], not to mention the negative effect of the vulnerability of the global food trade network [84,85]. Beyond a certain threshold of trade openness, food security status tends to improve. This indicates that relative price changes will finally result in changes in resources allocation on various sectors followed by changes in production’ subsectoral levels, and in turn, changes in income levels (which are expected to increase in aggregate as resources are used more efficiently) have the potential both to decrease poverty levels and improve food security by improving poor’s food acquisition [38]. Imported food supply—generally with relatively higher quality and cheaper prices—has intertemporal substitution effects or income effects, which means an increase in real income, and induces stronger domestic demand [86,87]. Therefore, participation in the world markets through international trade can finally improve food security in Central Asian countries.

The empirical findings in Table 5 further confirm that other economic factors and non-economic factors chosen as control variables in this study can also be important determinants of food security. Both GDP per capita and GDP growth have positive coefficients—most of which are statistically significant—indicating that sustained economic development is a major factor in improving food security in Central Asian countries. Employment in agriculture has negative coefficients and all of which are statistically significant, meaning that there is likely to be a labor surplus in Central Asian countries’ agriculture. Both arable land and freshwater withdrawals in agriculture have negative coefficients, most of which are statistically significant, indicating that the utilization efficiency of arable land and water is low in Central Asian countries’ agriculture, which is not conducive to improving food security in these countries. It has been calculated that the average consumption coefficients of arable land denoted by the ratio of arable land area and total cereal production in Central Asia are much higher than the world average value of up to 7.74 m^2^/kg, which is 3.6 times that of China, and the average agricultural water consumption denoted by the ratio of water used in agriculture and total cereal production in Central Asia is about 9.43 m^3^/kg, or nearly 9.3 times the average value elsewhere in Asia [88]. Agricultural productivity has positive coefficients, most of which are statistically significant, indicating that an increase in agricultural productivity is conducive to food security’s improvement. Increased agricultural productivity often means an increase in food production and supply capacity, and thus especially improving availability, access, and stability from a domestic food supply perspective. Population growth has negative coefficients, which shows that population growth is not conducive to the improvement of food security. A larger population always means greater food demand, which in turn increases the pressure to ensure food security. Natural disasters have negative coefficients, which suggest that natural disasters are not conducive to the improvement of food security. Natural disasters usually cause considerable damage to the production and transport of food [89,90], and then availability, access, stability, and utilization of food will all be adversely affected. The inflation rate has negative coefficients, most of which are statistically significant, showing that the inflation rate is not conducive to the improvement of food security. Most countries in Central Asia have experienced persistently unstable and high inflation since independence [91], which has worsened their food security status, considering that inflation often means a decline in real income levels and purchasing power, especially for low-income classes.

### 4.2. Robustness Test

For testing the robustness of estimation results of the panel data fixed effect model, an LS procedure for pooled data and a DPD analysis with the GMM approach are used in this study, simultaneously.

#### 4.2.1. Estimation Results of LS Procedure for Pooled Data

The number of observations analyzed in this study is not very large, and there may be complicated panel error structure problems in the observations’ data, such as synchronous correlation, heteroscedasticity, and series correlation [92]. For dealing with these problems effectively, Beck and Katz [93] developed the weighted panel corrected standard errors (PCSE) method. According to weight covariance processing methods’ differences, PCSE can be further divided into cross-section weights, cross-section seemingly unrelated regression (SUR), period weights, and period SUR [92]. After comparison, based on cross-section SUR, we found both of independent variables’ t-statistics and models’ R-squared statistics were generally larger than those based on the other three approaches, indicating a total fitting effect of cross-section SUR is optimal. Consequently, in this study, we used the weighted PCSE cross-section SUR approach to analyze the LS procedure for pooled data. According to DW-statistics’ values, AR terms with appropriate lagged periods are also supplemented for removing probable self-correlation problems. It is found that the effect direction and statistical significance of most independent variables’ estimated coefficients in Table 6 were totally the same as the panel data fixed effect model’s estimation results in Table 5, which implies that the LS procedure for pooled data supports the consistency and validity of the baseline model estimators.

#### 4.2.2. Estimation Results of DPD Analysis with GMM Approach

Table 7 presents the estimation results of DPD analysis with the GMM approach by using the two-step system-GMM. The results of the autoregressive (AR) errors test indicate that all *p*-values of the AR(2) errors test were significantly larger than 0.1, therefore, the null hypothesis of no autocorrelation of order 2 was not rejected; the results of the Hansen test indicate that all *p*-values were significantly larger than 0.1, therefore, the null hypothesis that the tool variables are exogenous as a group was not rejected; the results of the Sargan test indicate that all *p*-values were significantly larger than 0.1, therefore, the null hypothesis of over-identifying restrictions was not rejected. Therefore, the specification for DPD analysis with the GMM approach in this study is reasonable and valid and can be estimated by using the two-step system-GMM. It is shown that effect direction and statistical significance of most independent variables’ estimated coefficients in Table 7 were totally the same as the panel data fixed effect model’s estimation results in Table 5, which implies that the DPD analysis with the GMM approach supports the robustness of the baseline model estimators. At the same time, all the lagged dependent variables were positive with statistically significant, showing that food security’s four pillars change tardily over time and are determined by their previous conditions, which justifies the specification of the dynamic panel model and the adoption of the two-step system-GMM for estimating DPD analysis with the GMM approach.

## 5. Conclusions and Policy Implications

Using the 2001–2018 panel data of Central Asian countries, this study is the first to quantitatively estimate trade openness and other factors’ impact on food security’s four pillars (including availability, access, stability, and utilization) and traces a U-shaped (or inverted U-shaped) relationship between trade openness and food security by adopting a panel data fixed effect model as the baseline model, and then conducts a robustness test by using an LS procedure for the pooled data and a DPD analysis with the GMM approach, simultaneously.

Estimates from the panel data fixed effect model indicated a U-shaped relationship between trade openness and food security’s four pillars in Central Asian countries, which means that the initial stages of trade openness negatively impact food security; furthermore, beyond a certain threshold of trade openness, food security status tends to improve, therefore, the participation in the world markets through international trade can finally improve food security in Central Asian countries. The empirical findings also confirm that other economic factors and non-economic factors chosen as control variables in this study can be important determinants of food security. GDP per capita, GDP growth, and agricultural productivity have contributed to the improvement of food security; employment in agriculture, arable land, freshwater withdrawals in agriculture, population growth, natural disasters, and inflation rate have negative impacts on food security. Estimates from the LS procedure for the pooled data and the DPD analysis with the GMM approach indicated that estimates from the panel data fixed effect model were robust and valid.

According to the above research conclusions, we can develop the following policy enlightenments to improve food security status for Central Asian countries: first, the countries need to build more robust trade policies and food safety technical rules that adhere to relevant rules of the WTO, such as decreasing burdensome and intricate trade regulations, increasing awareness about food safety standards, and improving the infrastructure of food safety and sanitary and phytosanitary, in order to maximize the positive effects of trade openness on food security. Second, the countries need to further boost government governance reform, to ensure sustained and steady economic growth and avoid large fluctuations in economic growth and exchange rate. Great importance should also be attached to social protection and safety nets policies, such as cash transfer programs, and transfers and subsidization of food [70], which are very important for low-income classes in these countries when facing various shocks and crises, including the ongoing COVID-19 pandemic. Third, the countries need to improve domestic agricultural support policies, especially producer support for agricultural inputs [27], research and extension of agricultural technology, and construction of agriculture-related infrastructure, to ensure reasonable food self-sufficiency by improving the utilization efficiency of agricultural resources, increasing agricultural productivity, and enhancing the capacity of agricultural producers especially small-scale farmers to better respond to natural disasters and climate change. Forth, the countries need to be more proactive in integrating into main global and regional cooperative platforms to obtain more external support for improving their food security status, such as China’s Belt and Road Initiative (BRI) which covers resources of urgent need in these countries, including agricultural investment, agricultural technology transfer, and massive investment in infrastructure [94,95], and promote the interface between these countries’ national development strategies and policies and main cooperation platforms based on mutual benefit and win-win cooperation.

This study has important scientific value and practical significance. Our research represents an attempt to provide further empirical evidence on the vital unresolved issue at the country level of typical transition countries, which will contribute to the research progress on the relationship between reform of trade policy and improvement of food security, and by extending research to food security’s four pillars, our research is a significant improvement over past studies mainly using poverty indicators for food security. Our research findings provide scientific support for sustainable food system strategies in Central Asian countries and also provide valuable implications for other transition countries and developing countries facing the common difficulty of realizing the Zero Hunger goal by 2030.

Further research should address some limitations of this study. First, limited by the data availability, our analysis selected one indicator for each of food security’s four pillars in Central Asian countries. However, there are a series of indicators for denoting and interpreting each of food security’s four pillars in the FAOSTAT. Hence, future studies should build an indicator system to evaluate each of the four pillars of food security, and based on which, more comprehensive evaluation results can be obtained. Second, based on comparison and by referring to the practice of previous studies [14,36,43,47], the share of trade value (total trade volume in goods and services) over GDP has been employed to measure trade openness in this study. Tariff [96], and free trade agreements (FTAs) [97] are also used as proxies for trade openness in some literature and deserve further analysis of these alternative variables’ impact on food security.

## Figures and Tables

**Table 1:** Variables and their data sources.

Variables		Unit	Symbol	Data Sources
Dependent variable	Dietary energy supply	kcal/capita/day	*DES*	FAOSTAT
	Rail lines density	per 10^2^ square km of land area	*RLD*	FAOSTAT
	Food supply variability	kcal/capita/day	*PCF*	FAOSTAT
	Population using safely managed drinking water services	%	*PUS*	FAOSTAT
Core independent variable	Trade openness	%	*TO*	WDI
Control variables	GDP per capita	Constant 2010 USD	*GDPC*	WDI
	Employment in agriculture	%	*EA*	WDI
	Arable land	Hectares per person	*AL*	WDI
	Freshwater withdrawals in agriculture	%	*FWA*	WDI
	Agricultural productivity	kg per hectare	*AP*	WDI
	GDP growth	Annual %	*GDPG*	WDI
	Population growth	Annual %	*PG*	WDI
	Natural disasters	%	*ND*	EM-DAT
	Inflation rate	Annual %	*IR*	WDI

Note: *DES*—dietary energy supply; *RLD*—rail lines density; *PCF*—food supply variability; *PUS*—population using safely managed drinking water services; *TO*—trade openness; *TO*^2^—quadratic form of trade openness; *GDPC*—gross domestic product per capita; *EA*—employment in agriculture; *AL*—arable land; *FWA*—freshwater withdrawals in agriculture; *AP*—agricultural productivity; *GDPG*—domestic product growth; *PG*—population growth; *ND*—natural disasters; *IR*—inflation rate. FAOSTAT—Food and Agriculture Organization Corporate Statistical Database; WDI—World Development Indicators; GDP—gross domestic product; EM-DAT—Emergency Events Database.

**Table 2:** Variables’ descriptive statistics.

Variables	Mean	Standard Deviation	Minimum	Maximum
Dietary energy supply	2684.71	372.77	1945.00	3240.00
Rail lines density	0.56	0.31	0.20	1.60
Food supply variability	47.11	34.53	9.00	188.00
Population using safely managed drinking water services	63.83	14.95	37.00	94.80
Trade openness	84.33	30.82	29.75	146.66
GDP per capita	3325.87	3286.36	447.96	11,165.57
Employment in agriculture	34.63	10.82	15.77	58.69
Arable land	0.53	0.64	0.08	1.98
Freshwater withdrawals in agriculture	86.30	12.36	58.38	96.52
Agricultural productivity	2529.38	1166.92	804.10	4851.70
GDP growth	6.72	3.25	–0.47	14.70
Population growth	1.55	0.54	–0.17	2.82
Natural disasters	0.97	5.68	0.00	38.84
Inflation rate	13.95	11.33	–5.15	59.74

Note: GDP—gross domestic product.

**Table 3:** Correlation analysis results between independent variables.

Variables	*TO*	*TO* ^2^	*GDPC*	*EA*	*AL*	*FWA*	*AP*	*GDPG*	*PG*	*ND*	*IR*
*TO*	1.000	0.985	–0.264	0.361	–0.082	0.104	–0.347	0.107	–0.095	0.216	0.063
*TO* ^2^	0.985	1.000	–0.306	0.369	–0.143	0.151	–0.284	0.105	–0.058	0.219	0.078
*GDPC*	–0.264	–0.306	1.000	–0.614	0.848	–0.398	–0.677	–0.012	–0.236	–0.129	–0.205
*EA*	0.361	0.369	–0.614	1.000	−0.396	0.309	0.109	0.154	0.189	0.154	0.108
*AL*	–0.082	–0.143	0.848	–0.396	1.000	–0.152	–0.644	–0.003	–0.469	–0.094	–0.084
*FWA*	0.104	0.151	–0.398	0.309	–0.152	1.000	0.532	0.071	0.293	0.070	0.044
*AP*	–0.347	–0.284	–0.677	0.109	–0.644	0.532	1.000	–0.127	0.290	0.001	0.194
*GDPG*	0.107	0.105	–0.012	0.154	–0.003	0.071	–0.127	1.000	–0.056	–0.049	0.154
*PG*	–0.095	–0.058	–0.236	0.189	–0.469	0.293	0.290	–0.056	1.000	0.036	–0.106
*ND*	0.216	0.219	–0.129	0.154	–0.094	0.070	0.001	−0.049	0.036	1.000	0.034
*IR*	0.063	0.078	–0.205	0.108	–0.084	0.044	0.194	0.154	–0.106	0.034	1.000

**Table 4:** Results of Kao cointegration test.

Variables	ADF		Residual Variance	HAC Variance
	t-Statistic	*p*-Value		
*DES*, *TO*, *TO*^2^, *GDPC*, *EA*, *AL*, *FWA*, *AP*, *GDPG*, *PG*, *ND*, *IR*	–3.791 ***	0.001	14.932	65.142
*RLD*, *TO*, *TO*^2^, *GDPC*, *EA*, *AL*, *FWA*, *AP*, *GDPG*, *PG*, *ND*, *IR*	–3.034 ***	0.001	0.006	0.007
*PCF*, *TO*, *TO*^2^, *GDPC*, *EA*, *AL*, *FWA*, *AP*, *GDPG*, *PG*, *ND*, *IR*	–3.801 ***	0.0001	52.450	45.883
*PUS*, *TO*, *TO*^2^, *GDPC*, *EA*, *AL*, *FWA*, *AP*, *GDPG*, *PG*, *ND*, *IR*	–3.218 ***	0.001	0.556	1.001

Note: ADF—augmented Dickey-Fuller; *p*-value—value of probability; HAC—heteroscedasticity-autocorrelation consistent. ***—1% significance level.

**Table 5:** Estimation results of panel data fixed effect model.

Variables	(1): *DES*	(2): *RLD*	(3): *PCF*	(4): *PUS*
Constant	15.501 (1.160)	0.495 (0.481)	38.880 (0.952)	17.259 *** (6.738)
*TO*	–14.769 *** (–4.599)	–0.008 *** (–2.707)	–1.818 ** (–2.116)	–0.167 *** (–2.963)
*TO* ^2^	0.080 *** (5.038)	0.00001 ** (2.203)	0.007 * (1.965)	0.001 *** (3.191)
*GDPC*	0.013 ** (2.426)	0.0001 *** (4.829)	0.005 * (1.989)	0.002 * (1.904)
*EA*	–1.841 *** (–3.357)	–0.017 *** (–3.161)	–1.545 *** (–5.297)	–0.658 *** (–4.239)
*AL*	–8.064 * (–1.902)	–3.969 (–0.756)	–4.498 ** (–2.027)	–4.337 * (–1.834)
*FWA*	–1.873 *** (–3.227)	–0.002 (–0.191)	–5.160 (–1.310)	–1.024 *** (–3.674)
*AP*	0.061 * (1.874)	0.0001 (0.806)	0.008 ** (2.644)	0.002 *** (2.848)
*GDPG*	3.915 (0.755)	0.006 * (1.848)	0.336 (0.269)	0.085 * (1.917)
*PG*	–8.595 * (–1.912)	–0.031 (–0.773)	–9.509 (–0.525)	–0.612 (–0.488)
*ND*	–0.487 * (–1.906)	–0.0002 (–0.129)	–0.570 * (–1.910)	–0.018 (–0.362)
*IR*	–1.208 ** (–2.034)	–0.003 ** (–2.385)	–0.444 * (–1.882)	–0.012 (–0.327)
Cross-section fixed effects	Yes	Yes	Yes	Yes
Period fixed effects	Yes	Yes	Yes	Yes
R-squared	0.944	0.936	0.864	0.986
F-statistic	29.762 ***	26.093 ***	21.303 ***	125.039 ***
**Redundant Fixed Effect Tests**				
Cross-section chi-square	62.506 ***	137.896 ***	20.376 ***	74.972 ***
Period chi-square	47.892 ***	11.825 ***	23.503 ***	33.327 ***
Cross-section/period chi-square	76.625 ***	151.049 ***	42.727 ***	80.968 ***
**Hausman Test for Random Effect**				
Period chi-square	69.174 ***	74.263 ***	90.854 ***	86.721 ***

Note: Numbers in parentheses are t-statistics values. ***—1% significance level; **—5% significance level; *—10% significance level.

**Table 6:** Estimation results of LS procedure for pooled data.

Variables	(5): *DES*	(6): *RLD*	(7): *PCF*	(8): *PUS*
*TO*	–17.203 *** (–3.417)	–0.008 *** (–3.930)	–1.332 ** (–2.147)	–0.146 *** (–3.575)
*TO* ^2^	0.042 *** (3.743)	0.00003 ** (2.773)	0.002 * (1.866)	0.002 *** (4.625)
*GDPC*	0.026 * (1.816)	0.0001 *** (3.188)	0.003 * (1.894)	0.001 *** (5.215)
*EA*	–1.215 *** (–5.465)	–0.003 ** (–2.343)	–1.078 ** (–2.198)	–0.631 *** (–4.552)
*AL*	–9.963 ** (–2.145)	–3.146 (–0.703)	–2.851 (–1.106)	–6.992 ** (–2.160)
*FWA*	–2.853 *** (–3.961)	0.009 (0.810)	–1.654 ** (–2.162)	–0.830 * (–1.856)
*AP*	0.104 *** (5.341)	0.0001 (0.775)	0.013 *** (2.901)	0.003 *** (2.672)
*GDPG*	1.522 (0.524)	0.008 ** (2.308)	0.413 (0.797)	0.051 (0.779)
*PG*	–7.472 *** (–3.489)	–0.097 (–0.857)	–3.200 *** (–4.025)	0.149 (0.801)
*ND*	–0.094 * (–1.881)	–0.0002 (–0.195)	–0.214 (–0.855)	–0.020 (–0.635)
*IR*	–0.020 (–0.820)	–0.005 ** (–2.103)	–0.189 (–0.987)	–0.037 ** (–2.118)
AR(1)	1.690 (1.288)	0.783 *** (2.945)	1.035 (0.934)	1.014 (1.368)
AR(2)		0.223 (1.408)		
R-squared (weighted)	0.963	0.981	0.845	0.973
DW-statistic (weighted)	1.920	1.897	1.939	1.964

Note: Numbers in parentheses are values of cross-section SUR correction t-statistics; AR—autoregressive; DW—Durbin–Watson; ***—1% significance level; **—5% significance level; *—10% significance level.

**Table 7:** Estimation results of DPD analysis with GMM approach.

Variables	(9): *DES*	(10): *RLD*	(11): *PCF*	(12): *PUS*
*DES(-1)*	0.815 *** (3.623)			
*RLD(-1)*		0.794 *** (4.189)		
*PCF(-1)*			0.802 *** (4.351)	
*PUS(-1)*				0.739 *** (5.026)
*TO*	–1.518 *** (–4.129)	–0.002 *** (–3.871)	–1.109 ** (–2.117)	–0.109 *** (–3.214)
*TO* ^2^	0.029 *** (2.918)	0.00001 ** (2.252)	0.010 ** (2.183)	0.001 *** (3.826)
*GDPC*	0.076 * (1.923)	0.0002 ** (2.497)	0.001 * (1.918)	0.003 *** (4.724)
*EA*	–1.674 *** (–4.918)	–0.003 ** (–2.286)	–0.895 * (–1.903)	–0.386 *** (–3.951)
*AL*	–8.752 * (–1.909)	–2.742 (–1.158)	–5.652 (–0.970)	–3.283 ** (–2.160)
*FWA*	–2.257 *** (–9.184)	–0.013 (–0.635)	–2.034 (–1.562)	–1.232 * (–1.921)
*AP*	0.095 *** (3.706)	0.001 (0.548)	0.009 *** (2.781)	0.021 *** (3.056)
*GDPG*	1.652 (0.524)	0.013 ** (2.124)	0.643 (0.571)	0.104 (0.634)
*PG*	–4.673 ** (2.108)	–0.205 (–0.817)	–2.428 ** (–2.061)	–0.085 (–1.236)
*ND*	–0.094 (–1.076)	–0.0001 (–0.443)	–0.373 (–0.692)	–0.012 (–0.784)
*IR*	–0.011 (–0.593)	–0.003 ** (–1.982)	0.304 (0.758)	–0.026 ** (–2.205)
*p*-value of AR(1) Errors Test	0.056	0.017	0.024	0.019
*p*-value of AR(2) Errors Test	0.439	0.452	0.519	0.523
*p*-value of Hansen Test	0.723	0.845	0.906	0.894
*p*-value of Sargan Test	0.384	0.372	0.268	0.291

Note: Numbers in parentheses are t-statistics values; *p*-values—values of probability; AR—autoregressive; ***—1% significance level; **—5% significance level; *—10% significance level.

## Data Availability

The data presented in this study are available on request to authors.
